# Long-term effects of canine parvovirus infection in dogs

**DOI:** 10.1371/journal.pone.0192198

**Published:** 2018-03-16

**Authors:** Elena Kilian, Jan S. Suchodolski, Katrin Hartmann, Ralf S. Mueller, Gerhard Wess, Stefan Unterer

**Affiliations:** 1 Clinic of Small Animal Medicine, Centre for Clinical Veterinary Medicine, LMU Munich, Munich, Germany; 2 Gastrointestinal Laboratory, Department of Small Animal Clinical Sciences, Texas A&M University, College Station, Texas, United States of America; University of Kansas Medical Center, UNITED STATES

## Abstract

**Background:**

Canine parvovirus (CPV) is the most important viral cause of acute canine enteritis leading to severe damage of the intestinal barrier. It has been speculated that dogs might develop chronic disorders after surviving CPV infection. However, no studies regarding the long-term implications of CPV infection have been published to date. The aim of this study was to evaluate whether dogs that have survived CPV infection will have an increased risk for developing chronic gastroenteritis, atopic dermatitis, or cardiac disease.

**Methodology/Principal findings:**

Dogs that had been treated at the Clinic of Small Animal Medicine, LMU Munich, for CPV infection for which a follow-up of at least 12 months was available, were included in the study. Owners completed a questionnaire on the presence of chronic gastrointestinal and cutaneous signs, cardiac disease, and other potential disorders. An identical questionnaire was sent to owners of matched control dogs during the same time period. Seventy-one questionnaires of dogs with CPV infection and 67 of control dogs were analyzed. Significantly more CPV-infected dogs (30/71) compared to control dogs (8/67) had developed chronic gastrointestinal signs later in their lives (*P* < 0.001). No significant differences were observed regarding skin diseases (*P* = 1), cardiac problems (*P* = 0.160), or any other diseases (*P* = 0.173) later in life.

**Conclusions:**

Results of this study suggest that dogs that survive CPV infection have a significantly higher risk (odds ratio = 5.33) for developing a chronic gastrointestinal disease. Further prospective studies to identify the trigger for the development of chronic diarrhoea and possible targeted treatment strategies are needed.

## Introduction

Canine parvovirus (CPV) represents a common viral cause of acute enteritis in dogs [1–3]. As parvoviruses require cells with a high proliferation rate for replication they have a high affinity for the small intestine, bone marrow, and lymphatic tissues [4]. In puppies, CPV can also affect myocardial cells during the time of high cell turnover rate from the time of intrauterine development until up to the age of about two weeks [5, 6] leading to acute heart failure frequently resulting in sudden death within the first eight weeks of life [7–9]. Structural changes in myocardial tissue have been detected in puppies surviving acute CPV infection [7], however their clinical consequence is unclear. In the intestine, characteristic histologic findings of parvovirus enteritis include necrosis of the intestinal crypt epithelium, shortening or obliteration of villi, and dilation of intestinal crypts with necrotic cellular debris [2]. These changes as well as the haemorrhagic diarrhoea seen in these dogs are associated with destruction of the intestinal barrier. An intact intestinal barrier is crucial for the development and stimulation of the immune system and establishment of oral tolerance. Severe destruction of the intestinal barrier might lead to a higher risk for immunological diseases later in life [10].

Puppies with CPV enteritis in combination with neutropenia are prone to become septic. Therefore, aggressive intravenous, broad-spectrum, bactericidal antibiotic treatment is part of the therapy. In humans, recent studies have shown that early-life exposure to antibiotics is associated with an increased risk for the development of allergic disorders [11, 12]. Metadata analysis suggests an association between acute gastroenteritis and development of post-infectious irritable bowel syndrome in humans [13]. Taken together, there is reason to suspect that severe enteritis in combination with antibiotic exposure in early life might also predispose dogs to signs of chronic gastrointestinal disease later.

So far, information about long-term consequences of CPV infection is sparse, and only few studies in veterinary medicine address long-term effects of acute intestinal disorders in general. However, this knowledge would be important to establish long-term prognosis. Thus, the aim of this study was to evaluate whether dogs that survived a clinical manifestation of CPV infection prompting intensive antibiotic treatment have an increased risk for chronic gastrointestinal and skin disorders, such as food or environmental allergies, or cardiac diseases later in life.

## Materials and methods

### Study design

The study was designed as a prospective survey in combination with retrospective data acquisition. A questionnaire with 37 questions was sent to owners of dogs that had recovered from a clinical manifestation of CPV infection. For comparison, the same questionnaire was sent to owners of dogs that had never experienced clinical signs consistent with CPV infection. Dogs in the CPV and control group were identified by reviewing medical records from the Clinic of Small Animal Internal Medicine of the LMU Munich, Germany, from July 1997 to December 2013. Only dogs monitored for at least one year after presentation were included in the study. Before sending out the questionnaires, owners of dogs suitable for the study were contacted by telephone and asked for their willingness to participate.

This study was approved by the ethics committee of the Centre of Veterinary Medicine, LMU Munich, Germany (approval number: 96-25-09-2017).

### CPV group

Initially, 237 dogs suffering from a clinical manifestation of CPV infection were included. Diagnosis was based on typical clinical signs in conjunction with a positive faecal antigen ELISA, positive PCR of faeces, or virological detection of CPV in faeces by electron microscopy.

To detect possible correlations between chronic gastrointestinal problems later in life and the severity of the CPV infection, selected laboratory parameters from the clinical records of the time of acute CPV infection of the puppies were evaluated. For consistency the most significantly changed value (with respect to the reference range) of each parameter recorded during hospitalization was used (e. g., the lowest recorded albumin level). In addition, clinical parameters such as the presence of blood in faeces or vomit, the first day of voluntary food intake, additional endoparasite infestation, antibiotic treatment (number of different antibiotics multiplied by number of days applied), and duration of clinical illness were documented.

### Control group

For each dog included in the CPV group a suitable control dog of identical breed and similar age (date of birth ± one year) as the dogs from the CPV group was selected (irrespective of their gender). Dogs were chosen that were presented in the same year as the corresponding dog with CPV infection. Those control dogs were presented to LMU Munich for wellness exams or vaccination. If matching healthy control dogs to a specific dog with CPV infection were not available, patients that were presented to the clinic for any reason except for gastrointestinal, dermatological or cardiac disorders or severe illnesses were included as control dogs. Dogs that were treated with antibiotics were specifically excluded.

### Questionnaire

The questionnaire comprised five sections (S1 File). The first section contained general information, such as origin of the dog, feeding regime, vaccination history, and parasite prevention. The second section included questions regarding chronic gastrointestinal problems. Episodes of gastrointestinal signs that lasted longer than three weeks or recurrent episodes lasting longer than three days and not improving without therapy were defined to be chronic. To assess severity of the gastrointestinal signs, questions were based on the Canine Inflammatory Bowel Disease Activity Index (CIBDAI) [14]. Questions always referred to the episodes with the most severe signs of the chronic gastrointestinal disorder. The third section evaluated presence of chronic skin diseases and was designed to identify dogs with atopic dermatitis according to the Favrot criteria [15]. The fourth section contained questions concerning the diagnosis of any cardiac disease and their associated clinical signs. In the last section, questions about the development of any other chronic diseases were asked. The questionnaire was formulated in layman’s terms and easy to understand. The comprehensibility was tested beforehand in a randomly chosen group of 10 dog owners without medical background.

### Statistical analysis

The comparison of baseline data, such as gender, breed, and endo- and ectoparasites prevention, of the CPV and the control group and the comparison of binary data were performed using Fisher’s exact test. A Mann-Whitney *U* test was used for ordinal scale data, such as age at presentation, time of observation, and CIBDAI. To identify correlations between clinical and laboratory findings during CPV infection and later gastrointestinal disease, Spearman’s rank correlation and Mann-Whitney *U* test were used. Bonferroni correction for multiple comparisons was applied where appropriate. *P* values < 0.05 were considered statistically significant. Power analysis showed that a minimum of 60 patients in each group was needed to detect a clinically significant difference of 30% in the prevalence of chronic disorders later in life with *P* < 0.05 and a power of > 90%. The statistical analysis was performed using R.

## Results

### Comparison of CPV and control group

For final analysis, 138 completed questionnaires were available, 71 questionnaires from the CPV group and 67 from the control group, respectively.

The CPV group consisted of 24 mixed breed dogs. The remaining 47 dogs comprised 25 different pure breeds. Most common breeds were Labrador (n = 6) and Golden Retriever (n = 6). Median age at presentation with acute CPV infection was 12 weeks (range 5 to 357 weeks) and median time of observation was five years (range 1 to 13 years). At the time of data evaluation 56/71 dogs were still alive. All 71 dogs had been presented with acute, mostly watery diarrhoea, which was haemorrhagic in 29 cases and 64 dogs had additionally suffered from vomiting, which was haemorrhagic in eight dogs. Sixty-six dogs were treated with antibiotics (23 dogs only with amoxicillin/clavulanic acid, 37 dogs with two different antibiotics, four dogs with three antibiotics, and two dogs with four antibiotics).

Twenty-three dogs of the control group were mixed breed dogs. The remaining 44 dogs belonged to 22 different pure breeds. Most common breeds were Labrador (n = 6) and Golden Retriever (n = 6). Median age at presentation for those dogs was 27 weeks (range 5 to 697 weeks). With a median of five years (range 1 to 12 years) the time of observation of the control dogs was similar to that for the CPV group. At the time of data evaluation 55/67 dogs were still alive. The majority of the control group (43/67 dogs) was presented for wellness exams and vaccination. Eight dogs were presented due to respiratory diseases, six due to urinary tract diseases, and five due to the supposed intake of foreign body or intoxication without clinical signs. A single dog each was treated for lameness, stick injury, a wasp sting, unrest, and weakness, respectively. None of these control dogs were treated with antibiotics or showed signs of gastrointestinal, dermatological, or cardiac disease.

No significant differences between dogs of the CPV and the control group were found regarding breed, date of birth, gender, age at data evaluation, time of observation, and regular prevention of ectoparasites. Age at presentation was significantly younger and regular prevention of endoparasites more frequently used in dogs of the CPV group (Table 1).

**Table 1:** Signalement and basic information of dogs of the CPV and control group.

Parameter	CPV Group	Control Group	*P*-value
Gender	52% Male (n = 37; 2 neutered)	48% Male (n = 32; 2 neutered)	0.734
	48% Female (n = 34; 3 spayed)	52% Female (n = 35; 9 spayed)	
Breed	66% Purebred (n = 47) Labrador Retriever (n = 6) Golden Retriever (n = 6) French bulldog (n = 4) Chihuahua (n = 3) Maltese (n = 3) Rottweiler (n = 3) Yorkshire terrier (n = 2) German shepherd (n = 2) Munsterlander (n = 2) Poodle (n = 1) Miniature schnauzer (n = 1) Australian shepherd (n = 1) Jack Russell terrier (n = 1) Pekingese (n = 1) Maremma sheepdog (n = 1) Doberman pinscher (n = 1) Rhodesian ridgeback (n = 1) Miniature pinscher (n = 1) Coton de Tulear (n = 1) Pomeranian (n = 1) Pug (n = 1) Cocker spaniel (n = 1) Podengo (n = 1) Kangal (n = 1) Magyar Vizsla (n = 1)	66% Purebred (n = 44) Labrador Retriever (n = 6) Golden Retriever (n = 6) French bulldog (n = 4) Chihuahua (n = 3) Maltese (n = 3) Rottweiler (n = 3) Yorkshire terrier (n = 2) German shepherd (n = 2) Munsterlander (n = 2) Poodle (n = 1) Miniature schnauzer (n = 1) Australian shepherd (n = 1) Jack Russell terrier (n = 1) Pekingese (n = 1) Maremma sheepdog (n = 1) Doberman pinscher (n = 1) Rhodesian ridgeback (n = 1) Miniature pinscher (n = 1) Coton de Tulear (n = 1) Pomeranian (n = 1) Pug (n = 1) Cocker spaniel (n = 1)	1
	34% Mixed breed (n = 24)	34% Mixed breed (n = 23)	
Age at presentation (weeks)	Median 12 (range 5–357)	Median 27 (range 5–697)	< 0.001
Time span of observation (years)	Median 5 (range 1–13)	Median 5 (range 1–12)	1
Prevention of endoparasites	94% Regularly (n = 67)	79% Regularly (n = 53)	0.011
	6% No (n = 4)	21% No (n = 14)	
Prevention of ectoparasites	58% Regularly (n = 41)	64% Regularly (n = 43)	0.488
	42% No (n = 30)	36% No (n = 24)	

### Evaluation of the questionnaire

**Fig 1:**
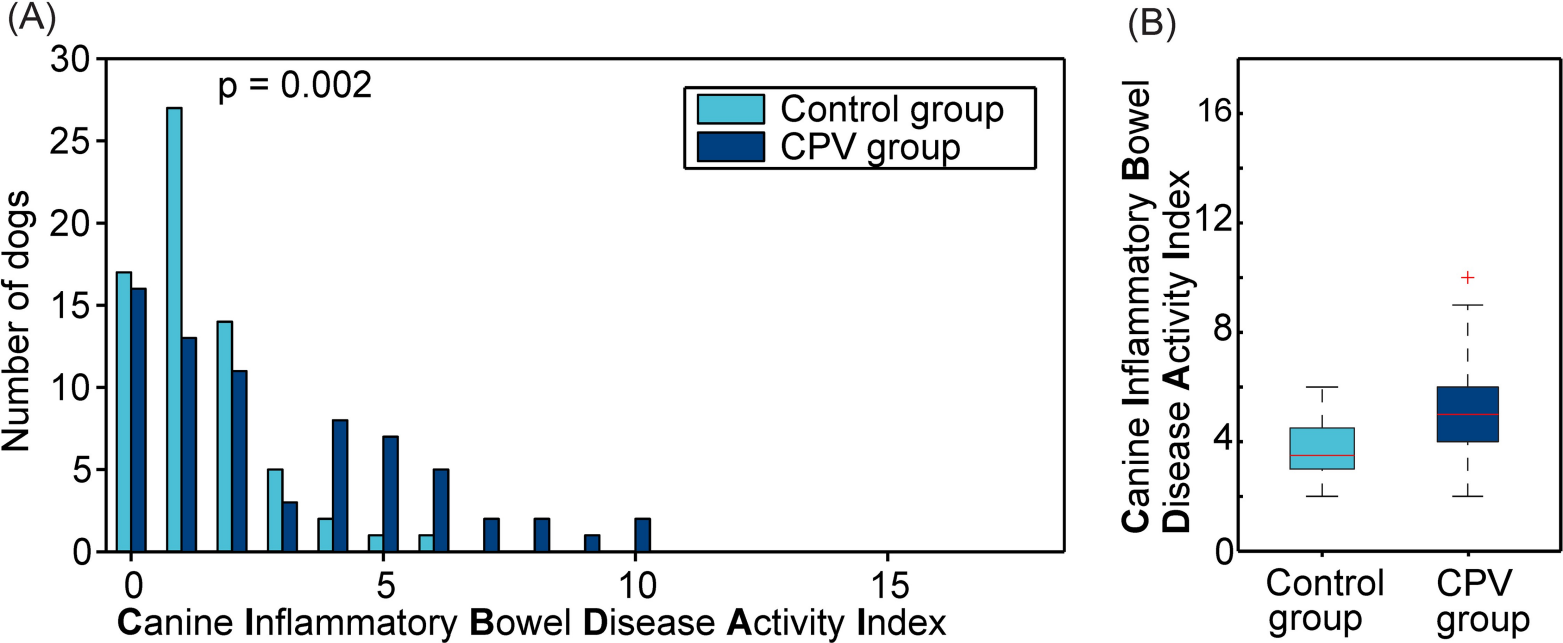
Panel A shows the distribution of the disease activity index CIBDAI (parameters activity, appetite, vomiting, stool consistency, stool frequency, and weight loss) of dogs of the CPV group (n = 71) and dogs of the control group (n = 67) referring to the episodes with the most severe signs of the chronic gastrointestinal disorder. Disease classification: 0–3: clinically insignificant disease, 4–5: mild IBD, 6–8: moderate IBD, > 9: severe IBD Panel B shows the comparison of both groups using a Box-Whisker-Plot.

Thirty of 71 owners (42%) of dogs of the CPV group reported chronic gastrointestinal problems compared to only eight of 67 owners of the control dogs (12%) (*P* < 0.001), showing a higher risk of chronic gastrointestinal problems following CPV infection (odds ratio = 5.33 [95% confidence interval = 2.12–14.87]). This was associated with a significantly higher CIBDAI in dogs of the CPV group (median 2, range 0 to 10), reflecting a higher disease activity, compared to the control group (median 1, range 0 to 6) (*P* = 0.002, Fig 1). When categorized in different CIBDAI severity groups (0–3: clinically insignificant disease, 4–5: mild, 6–8: moderate, > 9: severe disease), more dogs with previous CPV infection had mild (n = 15 vs. n = 3), moderate (n = 9 vs. n = 1), or severe (n = 3 vs. n = 0) disease compared to controls (*P* < 0.001). Stool consistency in dogs with prior CPV infection was significantly softer compared to controls (*P* < 0.001). Clinical signs of chronic gastrointestinal signs in dogs of the CPV group usually began during the first year of life (25/30; 83%), mostly with recurrent episodes (26/30; 87%). Chronic gastrointestinal signs responded to diet change alone in 19/30 dogs (63%). In 18/30 dogs (60%) gastrointestinal signs reoccurred throughout their lifetime whenever dietary or medical management was discontinued. Comparing dogs with chronic gastrointestinal signs of both the CPV (n = 30) and the control group (n = 8), no significant difference in severity of gastrointestinal signs, quantified by the CIBDAI, could be identified (*P* = 0.055).

The age at presentation with acute CPV infection varied from five to 357 weeks. To investigate possible correlations of the age of acute CPV infection to the prevalence of chronic gastrointestinal problems later in life, both question 2.1 and the CIBDAI index were separately analyzed for dogs of the CPV group split into two groups: No statistically significant difference was detectable between dogs ≤ 12 weeks compared to dogs > 12 weeks (*P* = 0.6344). Similarly, no difference was found for a threshold of 26 and 52 weeks, respectively (*P* = 1, *P* = 0.7498). Accordingly, the median age at presentation with acute CPV infection of dogs with chronic gastrointestinal problems later in life (n = 30; median age: 14.5 weeks) and dogs without chronic gastrointestinal problems later in life (n = 41; median age: 12 weeks) showed no statistically significant difference (*P* = 0.8196).

None of the laboratory or clinical parameters of the CPV group recorded during hospitalization were associated with an increased prevalence of chronic gastrointestinal disorders of the same dogs later in life (Table 2).

**Table 2:** Comparison of laboratory parameters.

Laboratory Parameters	CPV Group + GI	CPV Group—GI	*P*-value
WBC (10^9^/L)	5.01	5.50	0.215
Neutrophils (10^9^/L)	4.36	2.81	0.543
Lymphocytes (10^9^/L)	1.44	1.54	0.324
PCV (L/L)	31.42	31.50	0.758
Platelets (10^9^/L)	258.20	299.51	0.744
Total protein (g/L)	37.81	38,57	0.504
Albumin (g/L)	20.58	21.60	0.492

Comparison of the mean of selected laboratory parameters of dogs of the CPV group developing chronic gastrointestinal problems (CPV group + GI) and dogs of the CPV group not developing chronic gastrointestinal problems (CPV group–GI). For consistency the most significantly changed value of each parameter recorded during hospitalization was used. (Mann-Whitney *U* test)

Reference ranges: WBC 6–12 (10^9^/L), neutrophils 3–9 (10^9^/L), lymphocytes 1–3.6 (10^9^/L), PCV 35–58 (L/L), platelets 150–500 (10^9^/L), total protein 54–75 (g/L), albumin 25–44 (g/L).

Prevalence of skin diseases (*P* = 1), cardiac diseases (*P* = 0.160) or any other major diseases (*P* = 0.173) was not more common during lifetime in the CPV group compared to the control group (Table 3).

**Table 3:** Statistical evaluation of the main answers in the questionnaire (Fisher’s exact test).

Question about	Answer	CPV Group	Control Group	*P*-value
Chronic gastrointestinal problems	Yes	30 (42%)	8 (12%)	<0.001
	No	41 (58%)	59 (88%)	
Skin diseases	Yes	24 (34%)	23 (34%))	1
	No	47 (66%)	44 (66%)	
Cardiac diseases	Yes	10 (14%)	4 (6%)	0.160
	No	61 (86%)	63 (94%)	
Other diseases	Yes	31 (44%)	38 (57%)	0.173
	No	40 (56%)	29 (43%)	

## Discussion

The present study showed a significantly higher prevalence of chronic gastrointestinal signs in dogs that survived prior CPV infection. No evidence for a higher prevalence of cardiac or skin diseases or other severe illnesses was observed.

The exact prevalence of chronic enteropathies in dogs in general is unknown with manifold underlying causes for such signs [16]. According to the owners’ answers in the present study, most of the dogs improved with food change. Therefore, dogs with former CPV infection presumably suffer from food-responsive diarrhoea which includes immunological (food allergies) as well as non-immunological (food intolerances) causes [17, 18].

Potential reasons why chronic gastrointestinal diseases might occur after CPV infection are the CPV infection itself and the treatment of the parvovirus enteritis with antibiotics. As parvoviruses need cells with a high turnover rate, they especially replicate in the intestinal crypt epithelium and lymphoid tissues [6], which leads to severe destruction of the intestinal crypts and decreasing gut-associated lymphocytes followed by a destruction of the intestinal barrier. The destroyed gut barrier not only enables bacteria to translocate to the bloodstream but also hampers a physiologic development of oral tolerance allowing the penetration of macromolecules [19, 20]. Oral tolerance is characterized by lack of responsiveness of the immune system to certain antigens induced by prior oral administration of those proteins [19]. It is postulated that a breakdown in oral tolerance can result in food hypersensitivity [19], and this might represent one main mechanism for the development of chronic diarrhoea in dogs after CPV infection. Another potential impact is the development of auto-immunity following infection, as it has been shown that approximately 10% of patients suffering from an episode of acute self-limiting infectious gastroenteritis will develop chronic signs of diarrhoea predominant irritable bowel syndrome due to development of autoantibodies against intestinal tissue antigen [21].

There is increasing evidence that the balance between an intact commensal gut microbiota and the host defense at the mucosal barrier is important to prevent chronic intestinal inflammation. This has been shown in murine models, in which chronic colitis could be induced by changes in gut microbiota [22]. Acute diarrhoea per se, as well as antibiotics, a necessary standard therapy in CPV infections, cause a profound alteration of the intestinal microbiome [23]. Antimicrobials not only have a significant short-term impact on the microbiome, but even a long-term effect in some individuals [24]. In humans, it is known that early-life administration of antibiotics and the resulting changes in microbiota increase the risk of allergies, asthma, obesity, and IBD later in life [11, 25, 26]. It seems that the first six months of life is a ‘critical window’, during which an alteration of the microbiota might induce immunological events that promote allergic sensitization [27, 28]. Destruction of the epithelial barrier and alteration in the interaction between resident microbes and mucosa very likely play a pivotal role in the initiation and pathogenesis of chronic diarrhoea in dogs after CPV infection especially at an early age.

Osmotic diarrhoea resulting from damaged villi, which can stay shortened for the rest of the life, is another possible reason for the development of chronic diarrhoea. No histologic studies exist evaluating long-term morphologic changes of the intestinal mucosa after acute destruction due to CPV infection. Data about long-term mucosal changes in dogs surviving a CPV infection are not available for our patient population as well and should be collected in further prospective studies. Since most dogs with chronic diarrhoea after surviving a CPV infection responded to diet trials, no further diagnostic work-up was performed in those cases.

The absence of dermatological problems such as atopic dermatitis in dogs with prior CPV infection in this study is surprising. In humans, early-life exposure to antibiotics is associated with an increased risk of atopy later in life [29]. However, atopic dermatitis is a multifactorial disease in which a genetic predisposition as well as environmental factors play an important role [30, 31]. The pathogenesis and certain risk factors are not yet fully understood.

No long-term cardiac problems were detected in this study, although it is known that CPV infection can harm the myocardium and lead to structural changes in myocardial tissue [5, 7]. It seems that puppies having survived the acute damage of the myocardium die within a period of several weeks to months at the latest [32]. The absence of long-term cardiac effects might be explained by the fact that early CPV infection has become very uncommon as most adult bitches are vaccinated and therefore have CPV antibodies that lead to sufficient maternal antibody concentrations in puppies [2, 5].

This study shows that the development of chronic gastrointestinal signs seems not to correlate with the severity of the acute CPV infection as characterized by laboratory and certain clinical parameters.

Finally, it would be interesting to compare the prevalence of chronic gastrointestinal signs of dogs of the CPV group that received antibiotics to dogs of the CPV group that did not receive any antibiotics. However, due to the small number of dogs in the latter group (n = 5) no statistically meaningful evaluation can be performed.

The detection of risk factors for the development of chronic gastrointestinal problems is subject of further studies.

One limitation of this study is the reliance on subjective assessments of the dogs’ owners and the lack of standardization in types of examinations and diagnosing chronic gastrointestinal problems. It was tried to minimize this impact using standardized questions of the CIBDAI and criteria proposed by Favrot et al., which are validated scoring systems, in the questionnaire.

Another limitation is the small return rate of questionnaires that might be due to the fact that CPV infection is an acute illness for which puppies were treated in the clinic several years ago. Many affected puppies originated from animal welfare organizations. Their destination could not be retraced for data protection reasons.

It was not possible to prevent the control group from being significantly older than the CPV group due to the fact that it originated from the patient population of a clinic specialized in internal medicine where wellness exams and vaccination appointments are rarely performed. Furthermore, it was not possible to assure that control dogs did not receive antibiotics somewhere other than at our clinic for any reason. However, puppies that were presented for gastrointestinal problems were excluded from the control group to make sure no puppies with a CPV infection were included in the control group.

Therefore, an unambiguous correlation of a CPV infection with an increased risk for chronic gastrointestinal problems still cannot be drawn. Specifically, it might be possible that any severe acute gastrointestinal infection increases the risk for chronic diseases later in life as seen in humans [33–35]. Lacking any bacteriological or virological faecal examination (except for CPV) it cannot be excluded that some puppies had a coinfection, which might have had an impact on the clinical outcome. Future prospective studies are needed to differentiate between the influences of CPV infection itself, its treatment, or any other severe acute gastrointestinal insult acting as a trigger agent for chronic gastrointestinal problems. Investigations to prevent long-term effects in puppies with CPV infection such as probiotic therapy are strongly warranted.

## Conclusions

Dogs have a significantly higher risk of developing chronic gastrointestinal problems when having survived a clinical manifestation of CPV infection as puppy. However, the general risk for any other chronic diseases does not appear to be increased.

## Supporting information

S1 File Questionnaire. Questionnaire sent to owners of dogs that had recovered from a clinical manifestation of CPV infection and owners of dogs that had never experienced clinical signs consistent with CPV infection, for comparison.
(PDF)

S2 File Raw data. Data evaluated from the returned questionnaires including laboratory parameters noted during hospitalization of the dogs of the CPV group.
(XLSX)
