# Supplementary material for: Long-term effects of canine parvovirus infection in dogs
Source: PLoS One. 2018 Mar 16;13(3):e0192198. doi: 10.1371/journal.pone.0192198 (PMC5856261; doi:10.1371/journal.pone.0192198)
Supplement: S1 File — Questionnaire sent to owners of dogs that had recovered from a clinical manifestation of CPV infection and owners of dogs that had never experienced clinical signs consistent with CPV infection, for comparison. (PDF) [file pone.0192198.s001.pdf]

Part 1. Basic information

**1.1** Basic information	
Surname, Name<br><br>…………………………………………………………………………	
Your dog's name<br><br>…………………………………………………………………………	
Breed<br><br>…………………………………………………………………………	
Your dog's date of birth<br><br>…………………………………………………………………………	
Veterinarian in charge<br><br>…………………………………………………………………………	
Do you give the permission to contact your veterinarian in charge concerning laboratory findings/blood values/diagnostical findings?	
o Yes	o No

**1.2** Did you get your dog from a breeder?	
o Yes	o No
	Where did you get your dog from?<br><br>…………………………………………………………

**1.3** Has your dog always been living with you?	
o Yes	o No
	How old was your dog approximately when you got it?<br><br>…………………………………………………………

**1.4** Is your dog still alive?	
o Yes	o No
	When did your dog die/was your dog euthanized?<br><br>…………………………………………………………
	Was your dog euthanized?<br><br>…………………………………………………………
	What was the cause of death/euthanasia of your dog?<br><br>…………………………………………………………<br>…………………………………………………………

**1.5** How do you feed your dog? (dry food/canned food/self-cooked/barf feeding, which brand)?
....
....

**1.6** How did you feed your dog during its first year of life?
....
....

**1.7** Is your dog routinely vaccinated?	
o Yes	o No
When was the last vaccination?<br>...	

**1.8** Is your dog routinely dewormed?	
o Yes	o No
How often do you deworm your dog and when was the last deworming?<br>...	

**1.9** Is your dog preventively treated against ectoparasites (fleas, ticks) on a regular basis?	
o Yes	o No
How often do you treat your dog against ectoparasites and when was the last treatment?<br>...	

Part 2. Questions concerning a possible chronic gastrointestinal disease

Chronic gastrointestinal problems are defined as episodes with symptoms lasting more than three weeks or recurrent episodes of gastrointestinal problems that last each time more than three days or do not improve without therapy.
The questions 2.2 to 2.10 refer to these episodes of chronic gastrointestinal problems. If the severity of the symptoms varies, please refer your answers to the time of the worst symptoms ever occuring.

**2.1** Does/Did your dog suffer from chronic gastrointestinal problems?	
o Yes	o No
At what age did the symptoms occur the first time?<br><br>...	
How often does your dog suffer from gastrointestinal problems?	
o always	
o recurrent<br><br>mean duration of the episodes: ....<br><br>frequency of the episodes:	
o once from .... until	

**2.2** How would you describe the activity of your dog?
o normal
o slightly decreased
o moderately decreased
o severely decreased

**2.3** How would you describe the appetite of your dog?
o normal
o slightly decreased
o moderately decreased
o severely decreased
o increased

**2.4** How often does/did your dog vomit?
o 1 x/week
o 2-3 x/week
o > 3 x/week
o < 1 x/month

**2.5** How would you describe the stool consistency of your dog?
o normal
o slightly soft
o very soft
o watery

**2.6** How often does/did your dog defecate?
o normal
o 2-3 x/day or with mucus/blood
o 4-5 x/day
o > 5 x/day

**2.7** Does/Did your dog show a weight loss?
o No
o < 5 %
o 5-10 %
o > 10 %

**2.8** Has the albumin level of your dog been tested?		
o Yes:<br>o > 20 g/L<br>o 15-19 g/L<br>o 12-14 g/L<br>o < 12 g/L	o Unknown	o No

**2.9** Does/Did your dog suffer from ascites (free fluid in the abdomen) or oedemas (fluid retention)?
o No
o slight
o moderate
o severe

**2.10** Does/Did your dog suffer from itching additionally to the gastrointestinal problems?
o No
o occasional
o every day
o dog regularly wakes up because of itching

**2.11** Has your dog ever been examinated by ultrasound or endoscopy?	
o Yes (o ultrasound o endoscopy o both)	o No
What is the diagnosis?<br><br>..<br>..	

**2.12** Is your dog currently under therapy or has it been under therapy before?	
o Yes	o No
Did the symptoms subsequently improve/disappear?<br><br>o Yes o No	
What medication does/did your dog get?<br><br>..<br>..	

**2.13** Have you ever performed an elimination diet for your dog?<br>(Elimination diet is the exclusive feeding of only one source of protein (meat) and one source of carbohydrates that your dog has never eaten before in its life (commercially available diets, e.g. horse meat/parsnip, or self-cooked), for a period of at least 8 weeks, for ruling out or proofing of a food allergy)	
o Yes	o No
What did your dog get as elimination diet?<br><br>..<br>..	
Has it lead to an improvement?<br><br>o Yes o No	

Part 3. Questions concerning a possible skin disease

**3.1** Does/did your dog suffer from a skin disease?	
o Yes	o No
How was it diagnosed?<br><br>..<br>..	
What symptoms does/did your dog have?<br><br>..<br>..	
At what age did the symptoms occur the first time?<br><br>..	
Did the symptoms start before the third year of life?<br><br>o Yes o No	
Does/Did the complaints occur seasonal?<br><br>o Yes o No	
At which time of year are the symptoms the worst?<br><br>..	
Are/Were the front legs of your dog affected?<br><br>o Yes o No	
Are/Were the ear pinnae of your dog affected?<br><br>o Yes o No	
Are/Were the ear margins of your dog affected?<br><br>o Yes o No	
Is/Was the back part of your dog affected?<br><br>o Yes o No	

**3.2** Does/Did your dog have itching?
o No
o occasional
o every day
o dog regularly wakes up because of itching

**3.3** Does/Did your dog suffer from chronic or recurrent yeast or bacterial infections?<br>(o yeast infections o bacterial infections o both)		
o Yes	o Unknown	o No

**3.4** Is/Was your dog mostly indoor?	
o Yes	o No

**3.5** Have you ever performed an elimination diet for your dog?	
o Yes	o No
What did your dog get as elimination diet?<br><br>..<br>..	
Has it lead to an improvement?<br><br>o Yes o No	

**3.6** Have you ever performed an allergy test (skin test or blood test)?	
o Yes (o skin test o blood test o both)	o No
What is the diagnosis?<br><br>..	

**3.7** Is your dog currently under therapy or has it been under therapy before?	
o Yes	o No
Did the symptoms subsequently improve/disappear?<br><br>o Yes o No	
What medication does/did your dog get?<br><br>..<br>..	

**3.8** Did the itching disappear/improve during treatment with glucocorticoids (cortisone)?		
o Yes	o No	o Never got

Part 4. Questions concerning a possible heart disease

**4.1** Does/Did your dog suffer from a heart disease?	
o Yes	o No
How was it diagnosed? (auscultation, ECG, ultrasound)<br>……………………………………………………………………………………<br>……………………………………………………………………………………	
What symptoms does/did your dog have?<br>……………………………………………………………………………………<br>……………………………………………………………………………………	
At what age did the symptoms occur the first time?<br>……………………………………………………………………………………	

**4.2** Has an ultrasound of the heart been ever performed?	
o Yes	o No
When and where was it performed?<br>……………………………………………………………………………………<br>……………………………………………………………………………………	
What is the diagnosis?<br>……………………………………………………………………………………<br>……………………………………………………………………………………	

**4.3** Is your dog currently under therapy or has it been under therapy before?	
o Yes	o No
Did the symptoms subsequently improve/disappear?<br>o Yes o No	
What medication does/did your dog get?<br>……………………………………………………………………………………<br>……………………………………………………………………………………	

Part 5. Additional questions

**5.1** Does/Did your dog suffer from any other diseases during its life?	
o Yes	o No
What does/did your dog suffer from?<br><br>..<br>..<br>..	

**5.2** Did your dog suffer from any other diseases during its first year of life?	
o Yes	o No
What did your dog suffer from?<br><br>..<br>..<br>..	

**5.3** Is your dog currently under therapy or has it been under therapy before?	
o Yes	o No
Did the symptoms subsequently improve/disappear?<br><br>o Yes o No	
What medication does your dog get?<br><br>..<br>..	

**5.4** Does/Did your dog get medication for a longer period of time?	
o Yes	o No
What medication?<br><br>...<br>...	

Part 6. Further comments:

....<br>..<br>..<br>..<br>..<br>..<br>..<br>..<br>..<br>..<br>..<br>..
